# Access-Point Centered Window-Based Radio-Map Generation Network

**DOI:** 10.3390/s21186107

**Published:** 2021-09-12

**Authors:** Won-Yeol Kim, Soo-Ho Tae, Dong-Hoan Seo

**Affiliations:** 1 Department of Electrical and Electronics Engineering, Interdisciplinary Major of Maritime AI Convergence, Korea Maritime and Ocean University, Busan 49112, Korea; kwy00@g.kmou.ac.kr (W.-Y.K.); heavenroute124@gmail.com (S.-H.T.); 2 Division of Electronics and Electrical Information Engineering, Interdisciplinary Major of Maritime AI Convergence, Korea Maritime and Ocean University, Busan 49112, Korea

**Keywords:** access-point-centered window, adversarial learning, fingerprinting, radio-map generation network

## Abstract

Fingerprinting is the term used to describe a common indoor radio-mapping positioning technology that tracks moving objects in real time. To use this, a substantial number of measurement processes and workflows are needed to generate a radio-map. Accordingly, to minimize costs and increase the usability of such radio-maps, this study proposes an access-point (AP)-centered window (APCW) radio-map generation network (RGN). The proposed technique extracts parts of a radio-map in the form of a window based on AP floor plan coordinates to shorten the training time while enhancing radio-map prediction accuracy. To provide robustness against changes in the location of the APs and to enhance the utilization of similar structures, the proposed RGN, which employs an adversarial learning method and uses the APCW as input, learns the indoor space in partitions and combines the radio-maps of each AP to generate a complete map. By comparing four learning models that use different data structures as input based on an actual building, the proposed radio-map learning model (i.e., APCW-based RGN) obtains the highest accuracy among all models tested, yielding a root-mean-square error value of 4.01 dBm.

## 1. Introduction

Fingerprinting describes an indoor positioning technique that uses commonly available wireless area network technologies, such as wireless-fidelity (Wi-Fi), Bluetooth low-energy, and Zigbee [1,2,3] models. Numerous Wi-Fi fingerprinting studies have demonstrated improved transmission distances in indoor environments containing obstacles while supporting smartphone utilization. The fingerprinting process is normally divided into two phases: offline, which uses the Wi-Fi received signal strength indicator (RSSI), and online, which uses an established database to track the relevant positions in a real-time basis. In the offline phase, several reference points (RPs) are configured at normal intervals in the indoor area, generally divided into 2 or 3 m sectors that can be used to estimate a position based on the change of the measured RSSI [4,5,6]. Subsequently, Wi-Fi RSSI values for access points (APs) measured at all RPs are collected to build a radio-map database. Because the online phase attempts to estimate user positions based on similarities between the radio-map generated in the offline phase and the user’s RSSI values measured in real time, various probabilistic and deterministic algorithms can be utilized [6,7].

Furthermore, since the combined system can provide better accuracy than individual sensor systems, Poulose et al. [8] proposed a sensor fusion algorithm combined with a Pedestrian Dead Reckoning (PDR) system using an inertial sensor. In particular, it was demonstrated that the Wi-Fi fusion algorithm combining fingerprinting with the trilateration algorithm shows better positioning accuracy than the individual algorithms. Poulose et al. [9] proposed a hybrid deep learning model (HDLM) to improve location accuracy. The proposed algorithm proved that the combination of a convolutional neural network and long short-term memory network (CNN-LSTM) has better positional accuracy than other deep learning models. In particular, in order to solve the problem of RSSI, which can be easily changed according to indoor channel conditions, the location accuracy was improved by replacing the RSSI raw value with the RSSI heatmap. As such, the performance of a deep learning system depends on the quality of the feature representation, even with the same data. More recently, Ssekidde et al. [10] introduced novel feature set extractions based on the continuous wavelet transforms (CWT) of the received signal strength indicator’s (RSSI) data. CWT image-based feature sets have been demonstrated to improve performance by augmenting them with additive white Gaussian noise.

Such fingerprinting techniques require radio-maps for recognizing user positions, and the most basic method of generating a radio-map is point-by-point calibration, which measures the RSSI values of the APs at all RPs several tens of times over a certain period [11]. Doing so, the number of required RPs increases with the building size. In turn, such approaches require increased time and heavy costs. To address this issue, several studies have been conducted on walking surveys [12,13], crowd sourcing [14,15], and data augmentation [16,17]. A walking survey [18,19] is the most common RSSI measurement approach, and it directly measures the receiver strength while moving along a predetermined path. This method has the advantage of accurate data collection, but it has the disadvantages of long collection time and high workload. Crowd sourcing [20,21] makes use of random measurement traces collected by a user carrying a smartphone throughout the localization area. This has the advantage of easy updates; hence, it is used extensively. However, this approach is computationally complex and time consuming, and obtaining high accuracy is challenging. Data augmentation [22,23,24] is mainly used to address the measured RSSI data sparsity issue, which often limits the performance of neural-network classifiers.

Because RSSI is determined by complex indoor environmental factors, such as geometric (optical-based) propagation characteristics, some approaches to generating radio-maps using the physical dimension of a floor plan are underway to minimize the time-cost and workloads required [25]. There are two essential components of the database required for radio-map generation: the floor plan (location of the obstacle and AP) and the corresponding radio-map. Information related to the thickness or material of the obstacle is an additional condition needed to improve the accuracy of radio-map generation. Ali et al. [25] generated radio-maps by integrating path losses over certain trajectories with the floor plan/wall attenuation factor to make selections based on the thickness and material properties of the walls and obstructions. Zineb et al. [26] proposed a multi-wall and multi-frequency indoor path-loss prediction model using artificial neural networks to solve problems of model calibration and tuning using real measurements collected in a given environment at a set frequency. Mendoza-Silva et al. [27] proposed a new approach to handle sample collection using support vector regression for data enrichments. 

On the other hand, research on how to construct a radio-map based on a generative adversarial network (GAN), which uses unsupervised learning, is being studied. A GAN has the capability to provide excellent results in the field of image generation. Given enough training data, it is possible to emulate the data distribution in almost any image domain. This outstanding performance of GANs has been extended not only to the image generation field but also to other research fields. For example, Zhao et al. [28] proposed a GAN-based vehicle trajectory prediction method for urban roads. The discriminant network of a GAN consists of two independent networks, each receiving input and output data from the generated network to achieve better prediction accuracy.

Zou et al. [29] proposed a radio-map construction method that converges SLAM and a GPR-based GAN through a mobile robot to construct a detailed radio-map to estimate RSS values from new coordinates. This approach generates a precise radio-map by dividing the indoor space into a free space and a constrained space. Free space refers to an open space that mobile robots can easily access, such as a hallway or public space, and a constrained space refers to a closed space such as a private space or cubicle. Therefore, in free space, both floor plans and radio-maps are constructed using simultaneous localization and mapping (SLAM) based on data acquired by LIDAR and a Kinect Camera installed in the mobile robots. Constrained space cannot semantically collect RSSI values with the SLAM. Therefore, in the constrained space, information on a specific location is input into the GPR model trained with the RSSI values actually measured in the free space. The RSSI value in the constrained space can be estimated by inputting the output value of the GPR model to the generator of the GAN along with the noise. Therefore, in order to construct a precise radio-map, the performance of a GPR model trained with RSSI values collected through a mobile robot in free space is important. Seong et al. [30] used an unsupervised dual radio-mapping algorithm to generate a radio-map of an entire building based on the measured radio-map of one reference floor. Liu et al. [31] generated a desirable radio-map using an adversarial learning-based network from the accumulated indoor radio designed by human experts.

Most approaches to radio-map generation using floor plans have low versatility, owing to the size of the radio-map that can be generated based on the floor plan. Furthermore, datasets comprising various raw floor plans and radio-maps are required. A radio-map generation model trained on the basis of a floor plan must calculate not only the locations that can receive signals from the installed AP, but it also must anticipate locations that will not receive the signals. Finally, to represent obstacles as morphological two-dimensional (2D) images, they are converted into binary or gray scale. However, the binary scale cannot provide information about the propagation attenuation coefficient according to the obstacle’s material, and a gray-scale image requires additional propagation measurements and analyses for each material type as designed by experienced radio engineers.

Therefore, in this study, we propose an access-point centered window (APCW)-based radio-map generation network (RGN) to minimize costs and increase usability. The proposed model extracts the corresponding floor plan and radio-map information using the APCW, which is a window divided into the center of the AP location; thus, the radio-map generation accuracy improves by reducing the generation condition according to the location of the AP. The obstacles represented on the floor plan are converted into a one-hot vector to extract semantic features without additional propagation measurements or analyses, depending on the type of the material. The major contributions of this work are summarized as follows:

- The proposed APCW can reduce the size of the neural network model and improve the radio-map generation performance by dividing the collected floor plan and radio-map-based datasets into AP-based floor plan-based datasets.
- The proposed representation of obstacles using one-hot vectors can automatically infer the influence of the attenuation coefficients of materials without additional measurements.
- Unlike the floor plan-based RGN model, the input data structure is learned from the vector with the same location of the AP because it learns by dividing it into a certain window centered on the AP. Thus, the weight vectors of the layer, which were learned in the form of the existing floor plan, are learned in the form of radiation, as with the propagation model. The APCW-based RGN model is highly versatile because it can generate a radio-map regardless of the size of the floor plan.

The rest of this paper is organized as follows. Section 2 provides an overview and theoretical background, and Section 3 describes the proposed APCW-based RGN. Section 4 provides details of the experimental design and discusses the performance evaluation and comparison results of radio-map and localization. We present the conclusions and scope for future work in Section 5.

## 2. Materials and Methods

This section mainly outlines the relevant methods for fingerprinting and CGANs. The purpose of this section is to point out the functions of radio-maps used in fingerprinting to aid in the analysis and validation of the proposed algorithm. In addition, the overall structure and learning method of CGANs are discussed to explain the deep learning technique used in the proposed algorithm.

### 2.1. Fingerprinting

**Figure 1:**
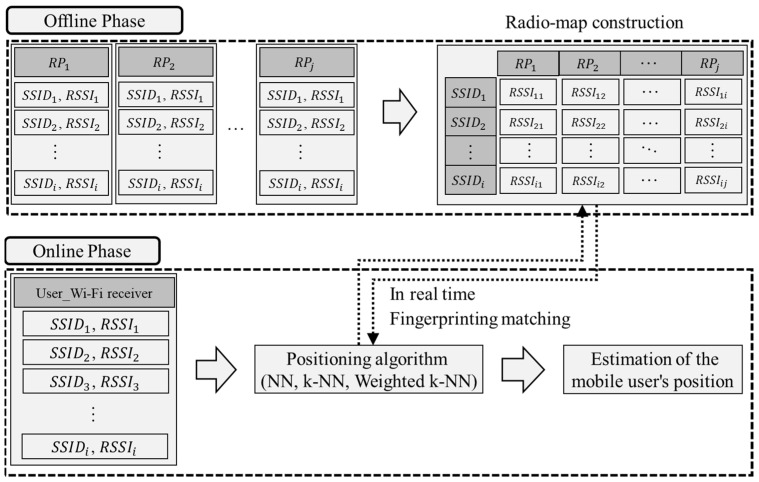
Structure of a typical fingerprint.

We describe the theory and approaches of a typical fingerprinting technique to highlight the importance of radio-map construction. Figure 1 illustrates a typical fingerprinting technique consisting of offline and online phases, as discussed. During the offline phase, a radio-map is generated by collecting the RSSI values of the visible APs at each RP. The service-set identifier represents the unique identifier of each AP. RSSI measurement is repeatedly performed at each RP for a sufficient number of repetitions to obtain a representative fingerprint value (e.g., via averaging [23]). The values are heavily influenced by relative distances and obstacle materials. However, with the increase of differences between the AP-measured RSSI values per RP, the accuracy of the position prediction increases.

The matrix of Equation (1) represents the structure of a typical radio-map. The *x*-axis denotes RPs; the *y*-axis represents the APs; and $RSSI_{SSID\left(i\right)RP\left(j\right)}$ represents the *RSSI* emitted from the *i*th AP measured at the *j*th RP.

(1) $$Radiomap=\left[\begin{array}{cccc} RSSI_{SSID1\left(RP1\right)} & RSSI_{SSID1\left(RP2\right)} & \cdots & RSSI_{SSID1\left(RPn\right)}\\ RSSI_{SSID2\left(RP1\right)} & RSSI_{SSID2\left(RP2\right)} & \cdots & RSSI_{SSID2\left(RPn\right)}\\ \vdots & \vdots & \ddots & \vdots \\ RSSI_{SSIDn\left(RP1\right)} & RSSI_{SSIDn\left(RP2\right)} & \cdots & RSSI_{SSIDn\left(RPn\right)} \end{array}\right]$$

During the online phase, the offline-generated radio-map is compared to the RSSI values of the APs measured from the user’s Wi-Fi receiver to predict their positions in real-time. Online, the RP having the highest similarity between the RSSI distribution collected in real-time and that of the constructed radio-map is used to estimate the mobile user’s position. Probabilistic or deterministic algorithms are utilized, because the position is estimated based on similarity [32,33]. For deterministic algorithms, the general form of position estimation is achieved by selecting RPs whose fingerprints are the closest match to the online *RSSI* measurements:

(2) $$P=\underset{j=1,\ldots ,N}{argmin}\sum_{i=1}^{M}d\left(RSSI_{ij},\;RSSI_{real}\right),$$

where $RSSI_{ij}$ is the representative fingerprint value for SSID i and RP j, and $d(RSSI_{ij},\;RSSI_{real})$ defines the typical distance metric. For time averaging, the representative value is the time-averaged fingerprint. Euclidean distance is a well-known distance metric for Equation (2), defined as

(3) $$d\left(RSSI_{ij},\;RSSI_{real}\right)=\|RSSI_{ij}-RSSI_{real}\|{}_{2},\;i=1,\ldots ,M.$$

A solution that finds the RP having the minimum Euclidean distance among measurements is known as the nearest-neighbor (NN) method. There are also various deterministic algorithms, such as k-NN [34], median filtering [35], and weighted k-NN [36], which can improve localization accuracy [37]. Moreover, there are various distance metrics, such as cosine similarity [38], Sorensen [39], and log-Gaussian [40], which can determine the accurate similarity between $RSSI_{ij}$ and $RSSI_{real}$. Localization performance is based on the premise that the radio-map contains representative values for each RP. Thus, radio-maps used for fingerprinting require considerable time and effort to produce representative values for each RP. Furthermore, increases in the size and complexity of building structures in recent years have significantly increased the amount of time and effort required to construct the radio-maps. Therefore, finding a method to reduce the amount of work and time is crucial.

### 2.2. Conditional GAN (CGAN)

**Figure 2:**
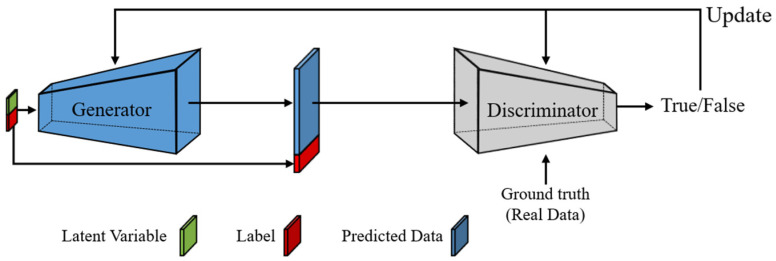
CGAN architecture.

A CGAN learns the distribution of real data that are difficult to predict using only modeling techniques, and it generates data that imitate the learned distribution [19,20]. Figure 2 illustrates the architecture of a CGAN comprising a generator and a discriminator. In the architecture, real data are ground-truth values that the CGAN attempts to imitate and generate using latent variables and labels. The latent variables denote the random variables that follow a normal distribution having a mean of zero and a standard deviation of one; this is used for training. The generator output data are considered predictions. The label represents a condition for generating real data and expresses auxiliary information (e.g., class). The CGAN adjusts the label as needed. 

The generator’s training process comprises three steps, as follows. First, the generator receives a latent variable and a label as input to generate the predicted data. Second, the discriminator attempts to distinguish real from predicted data and determine their similarity. If the predicted data are sufficiently similar to the real ones, the discriminator yields “True” (T); otherwise, it yields “False” (F). “True” indicates that the generator’s data generation performance is higher than the discriminator’s classification performance, whereas “False” indicates the opposite. Lastly, the generator and discriminator are trained according to the classification performance of the discriminator. The loss functions of the generator and discriminator for training are extrapolated, respectively, as Equations (4) and (5):

(4) $${ℒ}_{G}\left(x\right)=E\left[\log\left(D\left(G\left(v\right)|y\right)\right)\right],\;$$

(5) $${ℒ}_{D}\left(x\right)=E\left[\log\left(D\left(x|y\right)\right)\right]+E\left[\log\left(1-D\left(G\left(v\right)|y\right)\right)\right],\;$$

where v represents the latent variable; G and D denote the outputs of the generator and discriminator, respectively; x denotes the ground-truth value; and y represents the label. The key purpose of the CGAN is to generate a radio-map having an accuracy level that is indistinguishable from the real data. Training is carried out by minimizing the loss function of the generator, ${ℒ}_{G}$, while maximizing the loss function of the discriminator, ${ℒ}_{D}$. In the case of generating predicted data that are similar to real data using the trained generator, there is no need for a discriminator to evaluate the performance of the generator, because the main purpose is generation. Accordingly, during the generating phase, the discriminator that evaluates the generator’s performance is discarded, and the predicted data are generated using only the trained generator. 

## 3. Radio-Map Generation Network

### 3.1. Proposed RGN

**Figure 3:**
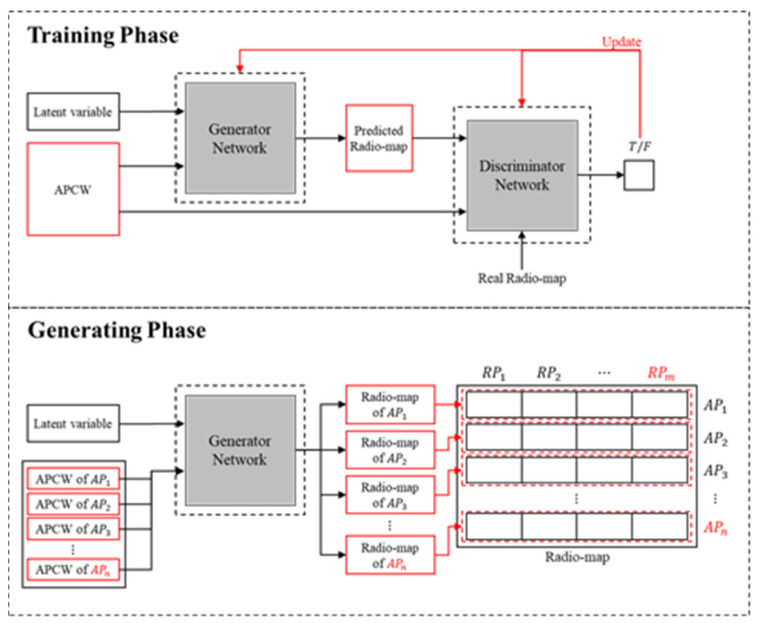
Architecture of the APCW-based RGN.

Figure 3 displays the architecture of the proposed APCW-based RGN. As with the CGAN, the proposed RGN comprises training and generating phases. During the training phase, the proposed APCW-RGN is divided into generator and discriminator networks. 

The generator network produces a radio-map by inputting a latent variable (i.e., a random variable following a normal distribution having a mean of zero and standard deviation of one) and the proposed APCW (i.e., label). The discriminator network calculates the probability of whether the input data is a real radio-map or a predicted (generated) radio-map under the APCW condition. Using this process, the generator network is trained iteratively using operation-loss functions (i.e., Equations (4) and (5)). During the generating phase, the discriminator network used in the training phase is removed, and only the generator network is used. Therefore, a radio-map is generated by repeatedly inputting the latent variable and APCW enough times to match the number of APs in the learned generator network. The following subsections introduce the APCW structural details reflecting the indoor environment and the RGN that predicts the RSSI values. Table 1 introduces the terms used in this work.

### 3.2. Access Point Centered Window

**Figure 4:**
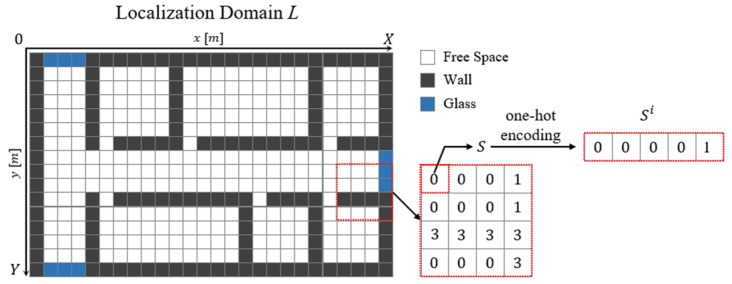
Structure of localization domain.

For RGN generation, it is necessary to reflect the physical environment (e.g., indoor layout and transmission distances) and the input structure of the label for training. To do this, we convert the indoor spaces into data to build the partitioned APCW centered around the AP. Figure 4 displays the localization domain after converting the entire floor plan into 2D data. Here, X and Y axes denote spatial distances, and the localization domain, L, comprises a set of cells that indicate certain spaces. Each cell is configured to a 1 × 1 m^2^ square in consideration of computing power and obstacle sizes that affect RSSI values within 3 m (a typical distance interval of Wi-Fi RPs). Accordingly, one cell is designed to contain spatial information S of one space, and localization domain L can be expressed by Equation (6):

(6) $$L=\left\{S_{\left(1,1\right)},\;S_{\left(2,1\right)},\;\ldots ,\;S_{\left(X,Y\right)}\right\},$$

where spatial information S  is given one index value according to an obstacle existing at arbitrary X and Y coordinates of L. 

Table 2 summarizes the index values of S according to material types. Index 0 is arranged to express inaccessible spaces for measuring the AP signal or spaces outside the building. Based on the L generation, the 2D indoor space can be converted into computer-processable data.

However, the input structure allows one cell to have two or more index values depending on the obstacle type, whereas the number of weights connected to the network is one. Thus, precise physical coefficients representing the material characteristics of different propagation media cannot be trained individually. To address this, each cell is vectorized by one-hot coding to enable one index to be connected to one weight. Equation (7) displays $L_{one-hot}$ and the vectorized L:

(7) $$L_{one-hot}=\left\{S_{\left(1,1\right)}^{i_{1}},\;S_{\left(2,1\right)}^{i_{2}}\;,\;\ldots ,\;S_{\left(X,Y\right)}^{i_{X\times Y}}\right\},\;S_{x,y}^{i}=\{\begin{array}{c} \begin{array}{c} 0,0,0,0,0,1\;\mathrm{if}\;i=0\\ 0,0,0,0,1,0\;\mathrm{if}\;i=1\\ 0,0,0,1,0,0\;\mathrm{if}\;i=2 \end{array}\\ \begin{array}{c} 0,0,1,0,0,0\;\mathrm{if}\;i=3\\ 0,1,0,0,0,0\;\mathrm{if}\;i=4\\ 1,0,0,0,0,0\;\mathrm{if}\;i=5 \end{array} \end{array}$$

Vector information $S_{x,y}^{i}$ is assigned a one-hot vector according to the index, i, of S, which exists. Additionally, information on the thickness of the obstacle is very important because it can have a great influence on significant propagation loss. Therefore, the thickness can be added to each cell by combining it with a one-hot vector on a gray scale. However, assuming that the thickness is constant for each material, the characteristics of the thickness can be semantically included in the one-hot vector. This enables the generation of a basic label structure that includes the building’s floor plan and position information affecting the RSSI. However, this approach does not enable partitioning L into useful parts, because it employs the 2D conversion of the entire building. This approach can generate radio-maps only in buildings having the same structural and obstacle characteristics. Furthermore, there are drawbacks of unnecessary computations within networks, which yield high RSSI prediction errors, because the radio-shadow areas where the AP’s RSSI values cannot be measured are also used as training input.

Owing to the path loss [41], the RSSI increases with locations near the AP, and it gradually decreases as the distance increases. Because RSSI has a small value at a location far from the AP, it is difficult to distinguish the cause of the attenuation effect of distances and obstacles. The floor plan-based RGN [27,30] consists of distance-feature extraction from AP and receiver locations according to the input floor plan and that of the environmental influence feature for conversion into RSSI. Hence, it is difficult to directly convey the environmental influence feature that determines the RSSI because a calculation process for implicitly extracting the distance feature is added.

**Figure 5:**
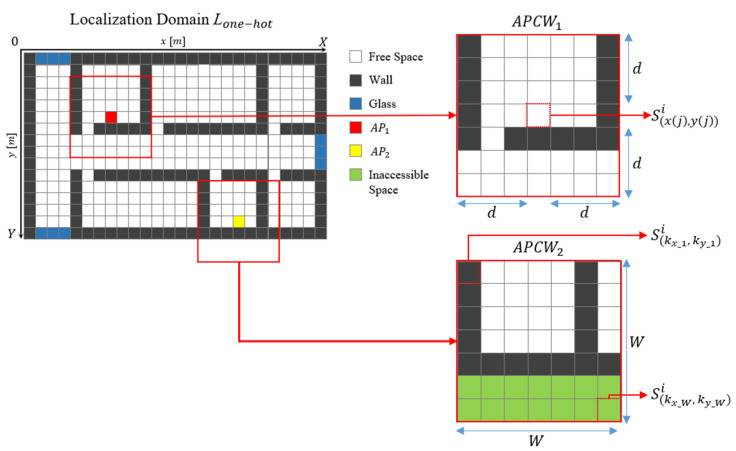
APCW structure.

The APCW converts the L of the floor plan using a window centered on the AP, so that each position of the input vector comprising the APCW includes distance information between the AP and the receiver. Therefore, the APCW-based RGN provides a clear generation condition for an obstacle or distance, because it is possible to directly infer a semantic correlation coefficient for an obstacle or distance than the floor plan-based RGN. Figure 5 illustrates the APCWs centered around the APs of two locations in $L_{one-hot}$:

(8) $$APCW_{j}=\left\{S_{\left(k_{x\_1},k_{y\_1}\right)}^{i},S_{\left(k_{x\_1}+1,k_{y\_1}\right)}^{i},\cdots ,S_{\left(k_{x\_W},k_{y\_W}\right)}^{i}\right\},\;$$

where $k_{x\_1}=x\left(j\right)-d$, $k_{y\_1}=y\left(j\right)-d$, and $k_{x\_W}=x\left(j\right)+d,\;k_{y\_W}=y\left(j\right)+d$. x(j) represents the x-axis coordinate of the jth AP; y(j) represents the y-axis coordinate of the same AP; d is the distance partitioned around the AP; $k_{x\_1}$ and $k_{y\_1}$ represent the first *X* and *Y* coordinates of $APCW_{j}$, respectively; and $k_{x\_W}$ and $k_{y\_W}$ represent the last *X* and *Y* coordinates of $APCW_{j}$, respectively. Thus, the generated $APCW_{j}$ is composed of W×W cells, and it can obtain obstacle information of a space that is d distance away from an AP. Here, because $APCW_{j}$ is generated based on the AP, location information of the AP is not used in the input. Furthermore, the size of network input data is $W\times W\times i_{n}$, which is the product of the APCW size, W×W, and the number of indices, $i_{n}$. Because the transmission distance at which the RSSI value is measured is generally determined centered around AP, the center of the window is configured as the AP’s location based on the root mean-square error (RMSE) between the predicted radio-map according to the APCW size and the real radio-map. The RMSE value represents the average difference between components constituting different data. The RMSE decreases as the distribution of the two data gets closer. Then, the highest RGN radio-map generation accuracy is obtained when the RMSE value between the predicted radio-map generated from the RGN and the real radio-map is the smallest.

Therefore, this study uses the RMSE value to determine the size of the partitioned squares optimized for an indoor environment. As such, although the RGN computational complexity is proportional to the number of indices, the proposed method of generating APCW centered around the APs can reduce the computational complexity of the network, because it does not involve adding indices to indicate the AP location information. An $APCW_{j}$ generated this way is used as a label in the generator to prevent overfitting for the entire building structure by reflecting the structural characteristics and obstacle material types while generating a desired region around the APs.

### 3.3. Radio-Map Generation Network Architecture

**Figure 6:**
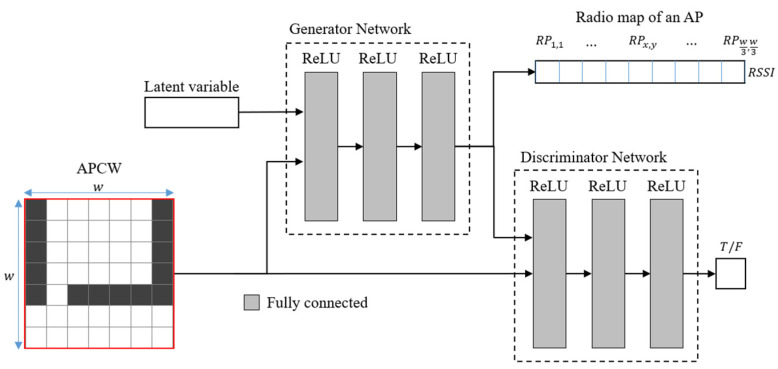
Structure of APCW-based RGN.

The RGN generator and discriminator were designed based on the CGAN. The generator provides labels for generating radio-maps using APCW-based input and output structures. Figure 6 illustrates an RGN structure comprising a fully connected (FC) layer-based generator and discriminator.

The input of the generator uses the latent variable, z, and the APCW for learning the distribution of the real radio-map. The CGAN generator uses a static-size transposed convolution. However, this is implausible in this case, because the measurable distance of the RSSI values distorted by surrounding obstacles centered around all APs in L is inconsistent. Accordingly, z and the APCW are used as input to the three FC layers having rectified linear units (ReLUs) as activation functions [42]. During this process, the ReLU activation function addresses the vanishing gradient problem of the generator and discriminator, which comprise three layers apiece.

Table 3 shows details of the generator’s layer type, its activation function, and its input and output sizes. In the table, z represents the latent variable size; $W^{2}$ represents the APCW size; and $i_{n}$ represents the number of indices needed to generate the APCWs. For CGAN network execution, the input and output sizes of the generator and discriminator should be accurately configured. Therefore, the input size of the generator is set as $z+W^{2}\times i$: the sum of the sizes of z and the APCW. The FC layer has inferior feature extraction performance compared with a convolution layer. Thus, the size of the first layer of the generator is configured as $2\times W^{2}$, which is larger than the APCW. This enables the extraction of as many APCW structural features as possible. Furthermore, the second and third layers are configured to have gradual decreases in size with $W^{2}÷3$ and $W^{2}÷9$, respectively, to predict RSSI values based on the features extracted from the first layer. Here, each component output size generated from the generator is set to 3×3 m, which is the typical interval used for measuring RSSI in fingerprinting. Because one component of the APCW expresses a 1×1 m space, the length and width of the radio-map output from APCW and the generator have a size ratio of 3:1. Accordingly, the length and width of the APCW should be set to at least 3 m. The radio-map generated by the generator after receiving $APCW_{j}$ as input is defined as $Predicted\;Radiomap_{j}$. 

**Figure 7:**
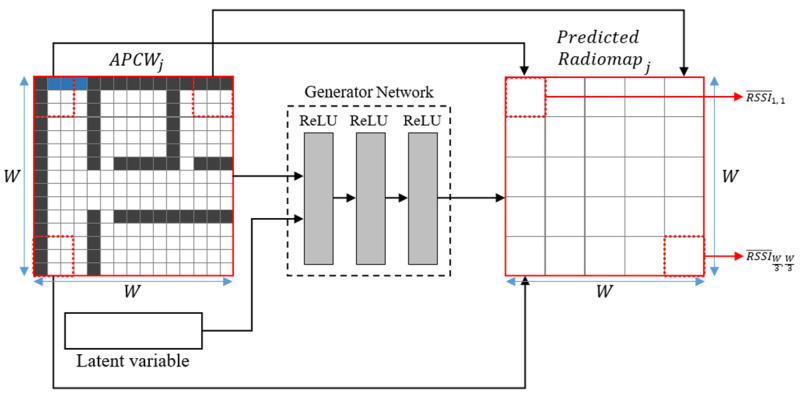
Structure of predicted radio-map.

Figure 7 displays its structure generated using $APCW_{j}$ and z as inputs. W denotes the diagonal entry size of the APCW. Each cell constituting $Predicted\;Radiomap_{j}$ is designed to predict an RSSI value of 3×3 m from the ACPW input to the generator. Thus, $Predicted\;Radiomap_{j}$ is composed of $\frac{W}{3}\times \frac{W}{3}$ cells (i.e., $\frac{1}{9}$ of the APCW). The generator uses the APCW as its input and is trained to output results that are like actual RSSI values. Therefore, $Real\;Radiomap_{j}$, which acts as ground truth for training, is a crucial factor for generator accuracy. The structure of $Real\;Radiomap_{j}$ is configured to be same as $Predicted\;Radiomap_{j}$ for an accurate comparison of RSSI values, and considering that RSSIs below −100 dBm are usually undetectable by most devices, we consider this value to be the lower limit of the accepted strength-value range [43,44]. To standardize the data, APs that are missing in a set of RSSI measurements will have their signal strengths = −100 dBm. Furthermore, the generated Real Radiomap is normalized to the range [0, 1] to increase the training speed of the network. Equation (9) expresses the normalization process:

(9) $${\bar{RSSI}}_{\left(x,\;y\right)}=\{\frac{RSSI_{\left(x,y\right)}-RSSI_{min}}{RSSI_{max}-RSSI_{min}},\;$$

where ${\bar{RSSI}}_{\left(x,y\right)}\;$ represents a value converted via the normalization of $RSSI_{\left(x,y\right)}$ (i.e., RSSI value measured at certain arbitrary *X* and *Y* coordinates). Additionally, $RSSI_{min}$ denotes the minimum RSSI value (i.e., −100 dBm) in $Real\;Radiomap_{j}$, whereas $RSSI_{max}$ denotes the maximum collected RSSI value. As the normalized value decreases, the signal strength increases. If the generator is trained with a sufficient normalized dataset, $\bar{RSSI}$, it yields an output value between zero and one. The final generated $Real\;Radiomap_{j}$ is provided as input for the discriminator alongside $APCW_{j}$.

The discriminator also comprises three FC layers that use ReLU as their activation functions to connect all components in the input layer. Table 4 shows details of the discriminator’s layer type, activation function, and input and output sizes. In the table, $W^{2}$ denotes the APCW size, and $i_{n}$ represents the number of indices used to generate the APCWs. The discriminator uses $Predicted\;Radiomap_{j}$, and $APCW_{j}$ (i.e., the label) are its input values. Thus, the input size of the discriminator’s first layer is set as the sum of the two input variables: $W^{2}÷9+W^{2}\times i_{n}$. Unlike the generator, the discriminator does not require layer scaling for feature extraction, because its main objective is to distinguish data. Accordingly, the discriminator’s layers are configured to have a gradual decrease in their output sizes with $W^{2}÷9$, $W^{2}÷27$, and 1, respectively. 

The output of the discriminator is either T or F because it determines whether $Predicted\;Radiomap_{j}$ is identical to $Real\;Radiomap_{j}$. Hence, the final output size of the discriminator is set to one. The proposed RGN outputs $predicticted\;Radiomap_{j}$ of an arbitrary AP, and the discriminator compares $predicticted\;Radiomap_{j}$ to $Real\;Radiomap_{j}$ to output their similarity. Subsequently, the CGAN-based network is trained using the discriminator’s output of similarity. Equations (10) and (11) are the loss functions applied to the generator and discriminator, respectively, for adversarial learning:

(10) $${ℒ}_{G}\left(z,L^{j}\right)=E\left[\log\left(D\left(G\left(z\right)|L^{j}\right)\right)\right],\;$$

(11) $${ℒ}_{D}\left(z,L^{j},R^{j}\right)=E\left[\log\left(D\left(R^{j}|L^{j}\right)\right)\right]+E\left[\log\left(1-D\left(G\left(z\right)|L^{j}\right)\right)\right],\;$$

where z represents the latent variable; G and D represent the output of the generator and discriminator, respectively; and $R^{j}$ represents the real radio-map of the jth AP. Additionally, $L^{j}$ denotes the APCW of the jth AP. The proposed network is designed to generate a radio-map having an accuracy level indistinguishable from actual data. Hence, the training process is conducted by minimizing the loss function, ${ℒ}_{G}$, of the generator while maximizing the loss function, ${ℒ}_{D}$, of the discriminator. The discriminator learns to maximize both the first and second terms in order to maximize Equation (11). That is, since $D\left(R^{j}|L^{j}\right)$ is the probability of classifying as a real radio-map, this output value should be as close to 1 as possible. Since $D\left(G\left(v\right)|L^{j}\right)$ is the predicted probability of classifying the radiomap, this output value should be as close to 0 as possible. Therefore, as the discriminator learns to maximize the value of the loss function, the discriminator learns to improve the performance of classifying whether the input radio-map is real or fake according to the given APCW condition. If the generator distribution is able to match the real data distribution perfectly, then the discriminator will be maximally confused, predicting 0.5 for all inputs. Based on the iteration of this training process, the generator’s output, $predicticted\;Radiomap_{j}$, gradually becomes identical to $Real\;Radiomap_{j}$. Subsequently, the generator can generate a radio-map of an AP using one operation when establishing radio-maps using the trained generator. Therefore, during the generating phase, one radio-map is generated for each AP.

**Figure 8:**
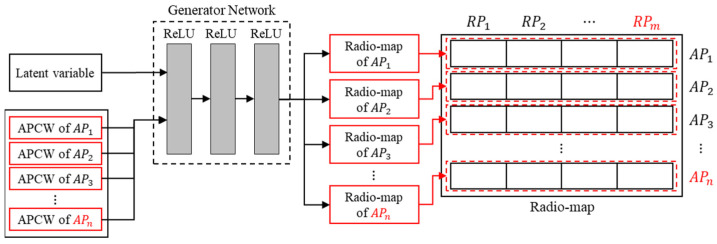
Radio-map construction process in the generation phase.

Finally, the generated radio-maps are combined to construe a single radio-map as shown Figure 8. In the figure, $AP_{n}$ represents the number of APs, and $RP_{m}$ represents the number of RPs. The output of generator is a value between zero and one. However, the unit of RSSI values measured in the online phase is in dBm units. Thus, the generator output is scaled via denormalization and input to the radio-map. The radio-map comprises a 2D matrix according to the AP and RP. Therefore, the predicted RSSI, which is a component of the predicted radio-map, is sequentially inserted by searching for the corresponding RP in the entire RP set. In other RPs, the minimum value of RSSI is inserted by assuming that the RSSI has a small value or that the location is not received. By repeating this process as many times as the number of APs, the entire radio-map of a 2D structure is constructed.

## 4. Experimental and Results

**Figure 9:**
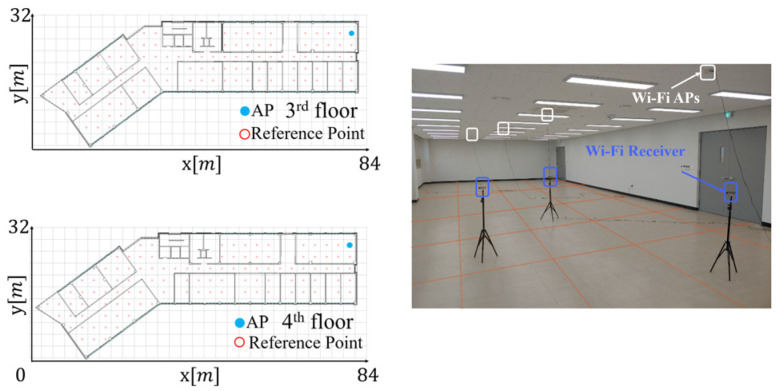
Experimental setup.

The radio-map generation accuracy of the RGN depends on the size of the APCW. Thus, an experiment was conducted to compare the radio-maps generated using various APCW sizes and to analyze the optimal APCW size. Additionally, four models using labels of different input structures were designed to analyze the effects of the proposed APCW on the RGN’s radio-map generation accuracy. The performance of the proposed APCW was verified by comparing the radio-map generation accuracy of each model. Subsequently, the characteristics of the networks trained with the proposed APCW were analyzed. To collect the Wi-Fi signals needed for the verification process, the experiment was conducted in a typical building environment, as illustrated in Figure 9.

### 4.1. Experimental Setup

In this study, to verify the validity of the proposed APCW-based RGN, we trained and validated the network using RSSI data collected in a real environment. For data collection, RSSI was measured on the 3rd and 4th floors of the Korea Maritime University’s Mieum Campus ($84\times 32{\;m}^{2}$). Figure 9 illustrate structures of the experimental space for each floor, including AP and Wi-Fi receiver installations. The RP consists of 222 APs total. There were 112 on the 3rd floor and 110 in the 4th floor with a measuring interval of about 3 m.

To build a sufficient dataset, the diversity of training data was increased by arranging APs for each RP and collecting RSSIs using the same AP (N3-i ipTIME N102-E) and Wi-Fi receiver (LG V20 smartphones) for signal uniformity. Because the CGAN used in the proposed network can include latent variables during the learning process, the RSSI measurement results can be used as training data. However, the RSSI average of all measurements per location was used to minimize uncontrolled and uncertain noise caused by extensive collection. Furthermore, the purpose of this study was to generate a radio-map of a similar area not measured through learning. Therefore, if a dataset includes various structural building characteristics, it will be possible to generate a radio-map for a space having structural similarity.

A desktop computer was used for verification of the proposed method and was equipped with Intel^®^ Core™ CPU i5-9400F, 32-GB memory, Geforce RTX 2070, 500-GB solid-state drives, and 2-TB hard disk drives. All proposed network learning was fixed at 1000 epochs to sufficiently converge and had a learning rate of 0.0001.

### 4.2. Effect of APCW Size on Network Performance

The proposed APCW was generated by partitioning $L_{one-hot}$ to a certain distance based on the AP location. During this process, if the distance being partitioned was shorter than the propagation distance of the AP, the RGN’s radio-map generation accuracy decreased. However, if the distance being partitioned was longer than the propagation distance of the AP, the RGN’s radio-map generation accuracy increased, because there was sufficient information provided. However, this resulted in increased time spent during RGN training.

**Figure 10:**
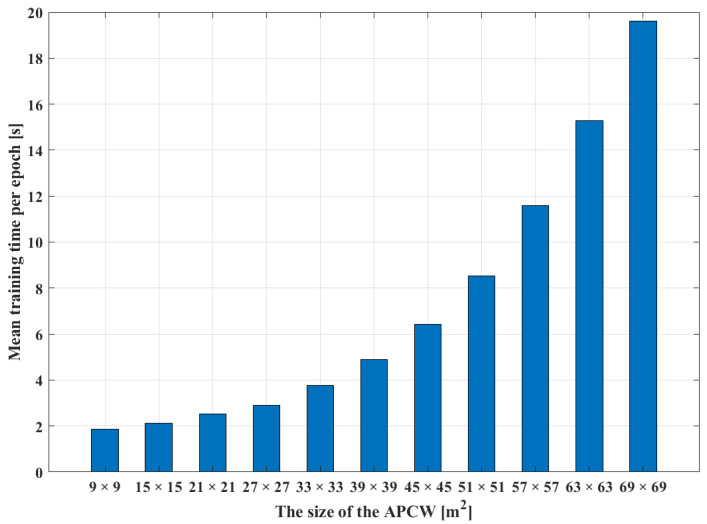
Mean RGN training time per epoch for varying APCW sizes.

In turn, the RGN’s radio-map generation accuracies according to APCW sizes were compared to derive the optimal partitioning distance. APCWs of different sizes were first generated at the same AP location. Subsequently, the generated APCWs were used as input to the RGN to compare the training times for each APCW size. Figure 10 illustrates the training times required per RGN epoch. The results were obtained by increasing the APCW size from $9\times 9{\;m}^{2}$ to $69\times 69{\;m}^{2}$. In the figure, the X-axis represents the size of the APCW, and the Y-axis represents the associated training time per epoch. The sizes of the APCWs used as input of the RGN were configured as multiples of 3 m, because each cell of the radio-map generated from the RGN expressed an RSSI of $3\times 3{\;m}^{2}$. Hence, the size of APCW was set to (1+2n)3 m×(1+2n)3 m. As shown in Figure 10, the training time of the RGN increased in proportion to the square of the APCW size. The training time increased significantly with the size of the collected database. Although both the RGN’s training time and the computational complexity decreased with smaller APCW size, the radio-map generation accuracy also decreased. Thus, it was necessary to consider both the RGN training time and radio-map generation accuracy when selecting the optimal APCW size.

**Figure 11:**
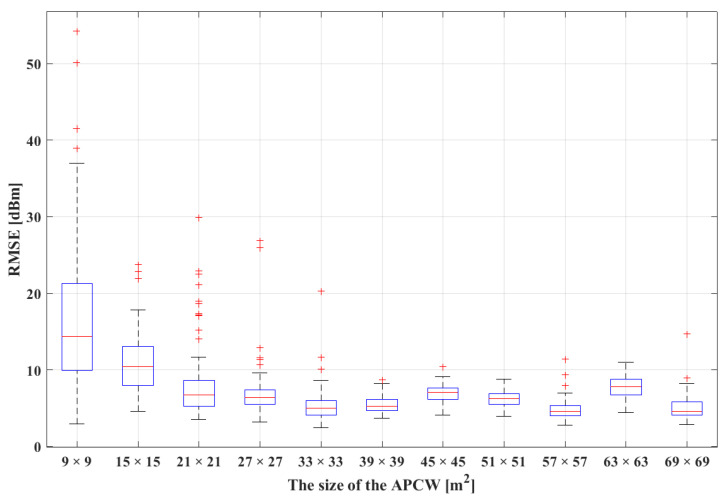
Boxplot of the RMSE distribution between the real and RGN-generated AP radio-maps by different APCW sizes.

In this study, the RMSE values were calculated using the predicted radio-maps generated from the RGN after using different sizes of APCW and real radio-maps. Then, the RGN’s radio-map generation accuracy was compared according to the size of the APCW. Figure 11 illustrates the distribution of RMSE values in comparison with the predicted and real radio-maps obtained by increasing the APCW size from $9\times 9{\;m}^{2}$ to $69\times 69{\;m}^{2}$. In the figure, the *X*-axis represents the APCW size, and the *Y*-axis represents the RMSE value. To statistically analyze the RGN radio-map generation accuracy, the *Y*-axis includes RMSE values of the predicted and real radio-maps calculated based on the AP locations. In the boxplot, a larger box representing the interquartile range (IQR) indicates a lower stability of radio-maps generated by the RGN due to the higher distribution of the RMSE. As the APCW size increased from $9\times 9{\;m}^{2}$ to $33\times 33{\;m}^{2}$, the median RMSE value decreased; additionally, the change in the latter was insignificant when the APCW size was larger than $33\times 33{\;m}^{2}.$ A smaller RMSE value indicates that the predicted radio-map generated by the RGN resembled the real radio-map more closely. An area of $33\times 33{\;m}^{2}$ was considered optimal because the median was the smallest among other APCW sizes. However, because the IQR and maximum RMSE were low, an area of $39\times 39{\;m}^{2}$ was the most stable RMSE. Additionally, during fingerprinting, the positioning accuracy decreased as the difference between the RSSI distribution collected in real time and the RSSI distribution stored in the radio-map increased. Changes in the RSSI distribution around the AP significantly reduced the positioning accuracy. Therefore, although the lowest median RMSE was obtained when the APCW size was $33\times 33{\;m}^{2}$, $39\times 39\;m^{2}$ was designated as the optimal APCW size when considering the IQR and maximum RMSE.

### 4.3. Generation Accuracy and Analysis

The proposed APCW was generated considering the obstacle types and materials of the indoor space and by removing the radio-shadow areas. Hence, the RGN using the proposed APCW generated accurate radio-maps. Four models using different labels as input were used to analyze the RGN’s radio-map generation accuracy according to the APCW.

Table 5 summarizes the descriptions, layer (activation function) types, and input and output sizes of the four models designed for the experiment. Model 1 is a basic RGN designed to compare its radio-map generation accuracy to that of the proposed APCW-based RGN, and it uses a label that reflects only the presence of obstacles as input, such as in [27,30]. Model 2 is an RGN model that uses a label reflecting only the material of obstacles as input. Model 3 uses a label based on the AP location while reflecting the presence of obstacles. Model 4 is the proposed APCW-based RGN that uses labels generated based on the AP location while reflecting the obstacle materials.

In the table, z denotes the latent variable; *X* denotes the size of the building along the *X*-axis; *Y* denotes the size of the building along the *Y*-axis; $W^{2}$ denotes the size of APCW; and $i_{n}$ denotes the number of indices. The generator was designed to receive a latent variable and a $1\times 1{\;m}^{2}$ label while generating a $3\times 3{\;m}^{2}$ predicted radio-map. Additionally, the discriminator receives the predicted radio-map generated by the generator alongside the label to determine whether the received predicted radio-map is identical to the real one. Because the input and output sizes of the generator and discriminator comprising each model were determined based on the size of the label, the input and output shapes of Table 4 are as follows. The label size of X×Y used as the input to Models 1 and 2 was set as $82\times 32{\;m}^{2}$ in accordance with the shape and size of the building. Furthermore, the label size of W×W used as the input of Models 3 and 4 was set as $39\times 39{\;m}^{2}$, which provides the optimal RGN radio-map generation accuracy according to the experiment results from Section 4.2. Meanwhile, the vanishing gradient problem can still be experienced because the generator and discriminator of each model comprise three layers each. To address this problem, ReLU was used as the activation function [42].

The Table 6 shows the network sizes of generators and discriminators according to each model. the size of the model changes according to the input structure of each model. When comparing Models 2 and 4, the size of the models shows a significant difference of about three times. When generating a radio-map, as the distance from the AP is increased, the distribution of the RSSI signal needs to be learned even in a space where the signal-to-noise ratio increases, so the radio-map generation accuracy can be reduced. In addition, floor plan-based RGN predicts not only the location that can receive the signal from the installed AP, but also the location where it will not receive the signal, so unnecessary computation is increased. On the other hand, since APCW-based RGN divides the radio-map prediction space into constant windows centering on the AP, it is possible to reduce the amount of unnecessary computation in the radio-map.

**Figure 12:**
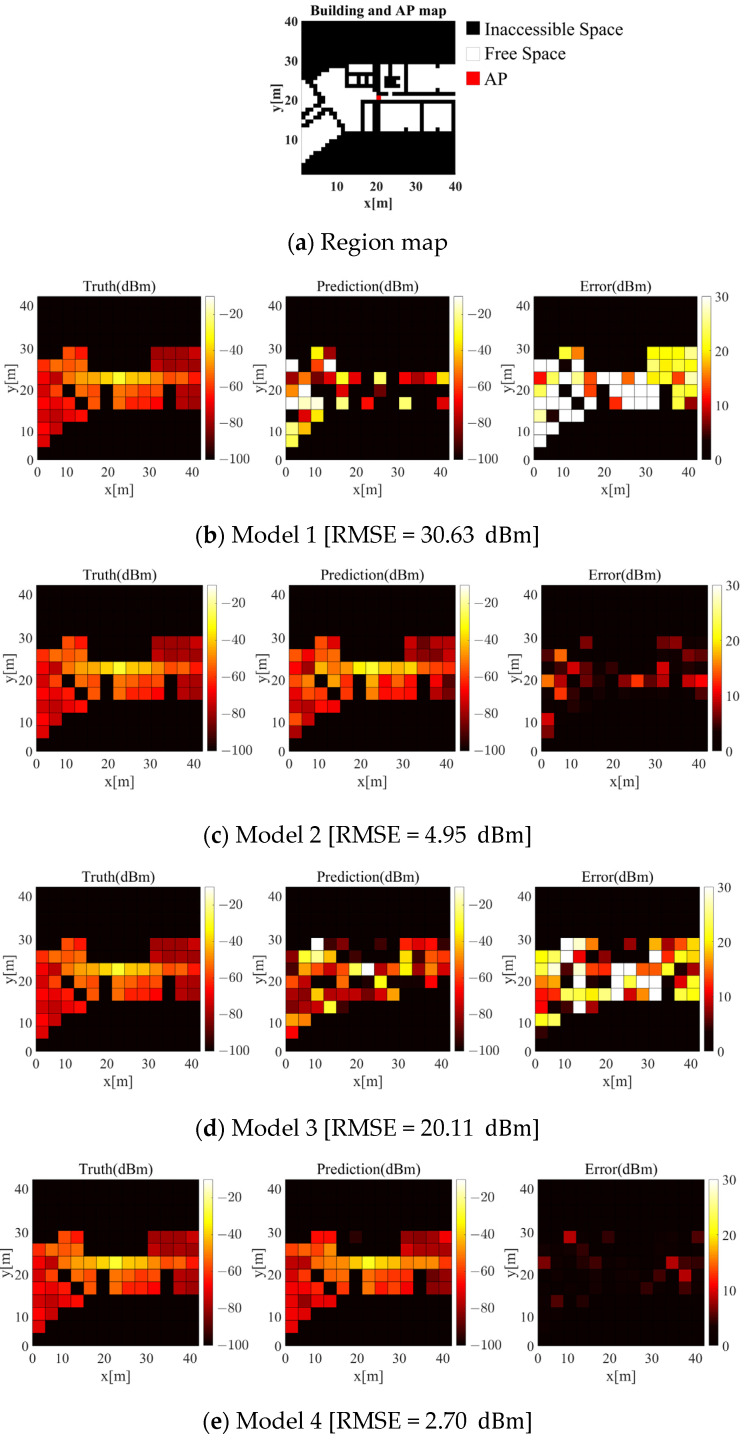
Model performance.

Figure 12 illustrates each model’s radio-maps generated by predicting the signals emitted from one AP. Figure 12a displays region maps to compare the generation accuracy of each model. In Figure 12a, red cells indicate the AP location; white cells indicate free space; and black cells indicate inaccessible space. Images in Figure 12b–e display the radio-map generation results obtained by Models 1, 2, 3, and 4. Models 1 and 2 generated $27\times 81{\;m}^{2}$ sized radio-maps using a label that converted an entire building map into data, whereas Models 3 and 4 generated $39\times 39{\;m}^{2}$ sized radio-maps. Hence, the size of the region-map used to compare the radio-map generation accuracy of each model was configured to $39\times 39{\;m}^{2}$, as with the output size of Models 3 and 4.

When comparing the results obtained by Models 1 and 2 and Models 3 and 4, it can be seen that the radio-map generation accuracy significantly increased when the obstacle materials were considered. This indicates that Models 2 and 4 learned that the degree of RSSI signal reduction from the AP differed according to the materials of obstacles (e.g., steel frame and concrete). In turn, the models predicted accurate radio-maps. Furthermore, as shown from the results of Model 4, which reflects both obstacle material and AP location, a high accuracy of radio-map generation was obtained at the center area adjacent to the AP. Accurate prediction of the radio-map of the area adjacent to the AP is important when maintaining the stable positioning accuracy of fingerprinting. As such, the advantages of Model 4 can be maximized, because it enables accurate prediction of the radio-map of the area adjacent to the AP. Images of Figure 12 represent radio-maps generated for one AP location point, and the accuracy can vary depending on the AP location. Therefore, to quantitively evaluate the radio-map generation accuracy of each model, radio-maps were generated for 110 different AP install locations, and RMSE values were derived. 

Table 7 displays the average, maximum, and minimum RMSE values obtained. As shown from the results of Models 1 and 3, the average RMSE value improved from 28.84 to 14.98 dBm when the label reflecting the AP location was used. Generally, a Wi-Fi signal has a standard deviation of ~0–5 dBm [45]. Accordingly, the radio-maps generated by Model 3 cannot be applied to fingerprinting, because the positioning accuracy is lower, owing to outliers. Thus, Model 3, which considers only the AP location, is unsuitable. Therefore, the radio-map generated by Model 3 considering only the location of the AP showed higher performance than did Model 1, because it learned the pattern of radio waves radiated to the AP location. When comparing the results of Models 1 and 2, the average RMSE significantly improved from 28.84 to 5.48 dBm when the label reflecting only the obstacle material was used. Thus, it can be inferred that the accuracy of RGN’s radio-map generation can be significantly improved, even when considering only the obstacle materials. 

Lastly, when comparing the results of Models 2 and 4, the accuracy of the RGN radio-map generation improved from 5.48 to 4.01 dBm when both the obstacle material and AP location were considered. As described in Table 5, Model 1 and Model 2 use the entire floor plan without dividing the window through APCW. The reason for this is that the loss function is learned not only in the close range of the AP, but also in the range that is difficult to predict, so the radio-map generation performance is comparatively lower than that of Models 3 and 4. Therefore, performance is improved by limiting the range away from the AP through APCW. Accordingly, highest radio-map generation accuracy was obtained when the proposed APCW was used as input to the RGN, and the average RMSE value was ~4 dBm, showing stable performance. As a result, Model 4 is the optimal RGN.

**Figure 13:**
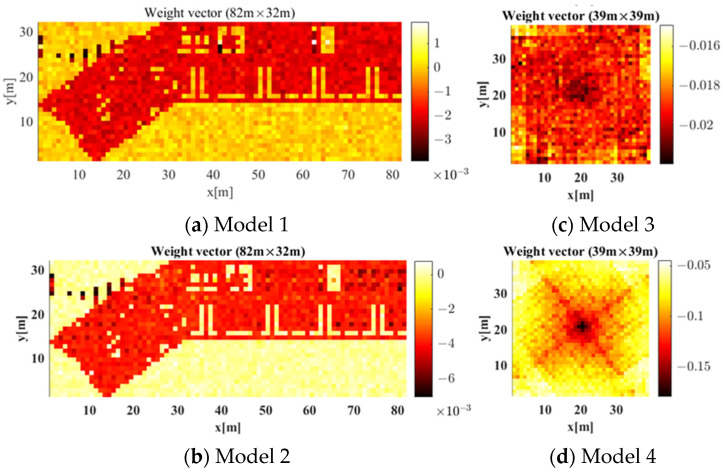
Weight Vectors of Model 1 to 4.

The radio-map generation accuracy was enhanced when the label reflected both obstacle material and AP location rather than only the material. The weight vectors constituting the input layers of Models 1 to 4 were compared to analyze the factors affecting radio-map generation accuracy. Figure 13 displays the weight vectors constituting the input layers of Models 1 to 4. In Figure 13a,b, *X* and *Y* axes represent *X* and *Y* coordinates of the weights constituting the weight vectors of Model 1 and 2. Here, the size and shape of the label were configured to be same as the size of the building. Additionally, in Figure 13c,d, *X* and *Y* axes represent *X* and *Y* coordinates of the weights constituting the weight vectors of Model 3 and 4. Here, the size and shape of the APCW were set as $39\times 39{\;m}^{2}$ (i.e., the size yielding the most stable radio-map generation accuracy of RGN based on the experiment results from earlier section). When data were entered into a typical network, each component constituting the data and the weight vector of the input layer were multiplied. Then, all multiplied values were added and sent to the subsequent layer. Thus, the weights for each location in the weight vector indicate the ratio of components used at the corresponding location in the input data to derive output results for the network.

As shown in the results of Model 1 and 2 in Figure 13a,b, the weight vectors were learned using the shape of the building as input, and the weights had similar values regardless of location, except for inaccessible spaces. Based on this, Model 1 and 2 used each component constituting the label similarly regardless of the distance between the component and the AP location when generating a radio-map. The signal strength emitted from the AP decreased as the distance from the AP increased, owing to path loss. This results in a radio-shadow area. However, Model 1 and 2, which show similar weights regardless of location, do not reflect a radio-shadow area. Thus, they cannot generate an accurate radio-map.

On the other hand, as shown in the results of Model 3 and 4 in Figure 13c,d, the weight vectors had the highest values at the center, which gradually decreased as the distance increased from the center. For signals, propagation phenomena (e.g., reflection and diffraction) occurred with obstacles, including walls, and the radio-map distribution changed, owing to the propagation phenomenon becoming smaller as the signal strength decreased. Accordingly, the radio-map distribution change became smaller with the increase of the distance between the AP and the obstacle.

Although the experimental environment does not resemble a perfect propagation pattern, because it used an indoor environment with various obstacles, Model 4 can more accurately learn these propagation characteristics than Model 3. Therefore, Model 4, which uses an APCW-based RGN to reflect both radio-shadow areas and propagation characteristics, enables the generation of accurate radio-maps.

**Figure 14:**
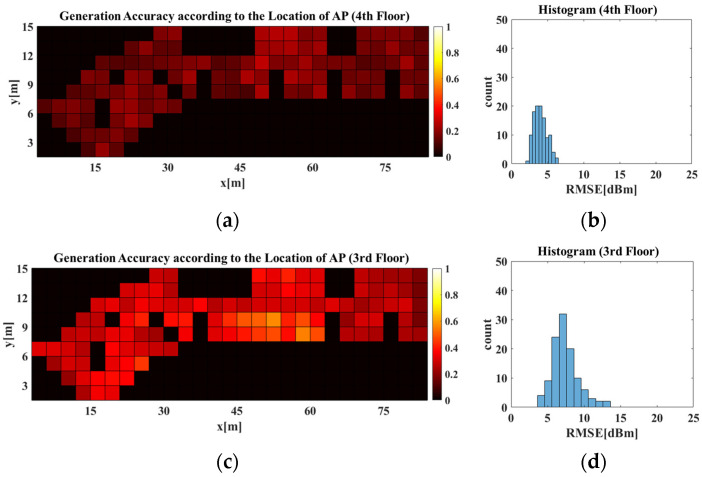
Generation accuracy of predicted (**a**,**b**) 4th and (**c**,**d**) 3rd floor radio-map using the RGN trained with 4th floor dataset.

To analyze whether the RGN that learned the indoor structural patterns generated accurate radio-maps, an experiment was conducted to compare the RGN accuracy per AP location. Figure 14 displays radio-map generation accuracies obtained from different AP locations on the 3rd and 4th floors using the RGN trained with data collected from the 4th floor. In Figure 14a,c, RMSE values between the predicted radio-map generated by the RGN when the AP were located at arbitrary *X* and *Y* coordinates in L, and the real radio-map of the corresponding AP location was displayed. Here, *X* and *Y* axes represent the coordinates of the AP location. Each cell shows the result of the RMSE of real vs. predicted RSSI in RPs according to the location of the AP, and the darker the color of each cell, the lower the error of predicted RSSI at the coordinates of the AP location.

**Figure 15:**
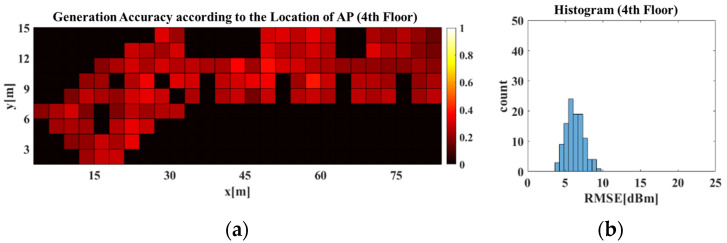

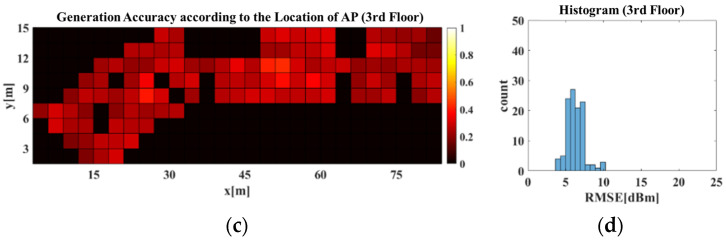
Generation accuracy of predicted (**a**,**b**) 4th and (**c**,**d**) 3rd floor radio-map using the RGN trained with 3rd and 4th floor dataset.

Figure 14b,d each show histograms of Figure 14a,c, respectively. A histogram expresses the size of components comprising the data and the frequency distribution of such sizes. Because there was difficulty checking results of individual cases, owing to the substantial number of APs and RPs in the experiments, histograms were used to statistically analyze the results. A smaller RMSE value indicates a higher radio-map generation accuracy. Thus, having a higher distribution on the left side of the RMSE histogram indicates higher radio-map generation accuracy. In the histogram, the *X*-axis represents the RMSE value between the predicted and real radio-maps, whereas the *Y*-axis represents the frequency of the values. As shown in the results of Figure 14a,b, the RGN generated an accurate radio-map regardless of the AP location on the 4th floor. This indicates that the RGN generated the radio-map after learning the structural patterns of the APCWs, which change per AP location and the radio-map distributions of the corresponding structures. For the results of Figure 14c,d, the RGN’s radio-map generation accuracy decreased on the 3rd floor compared with 4th floor when the AP was positioned at the center. This can be attributed to the structural difference at the center and the lower-left parts of the floors. Extant methods were limited to specific buildings used for training, but the proposed APCW-based RGN enables radio-map generation without measuring Wi-Fi signals, even when applied to new buildings. Thus, radio-maps were generated that learn all 3rd and 4th floor structures. Figure 15 displays the radio-map generation accuracy for the 3rd and 4th floors obtained using the RGN trained with data collected from 3rd and 4th floor dataset. where X and Y axes in the images of Figure 15a–d were the same as in the images of Figure 14a–d. As shown in Figure 15c,d, the RGN trained using data from both floors generated a more accurate radio-map compared with the RGN trained with data of only the 4th floor. This indicates that the APCW-based RGN learned patterns of new structures and generated a good radio-map.

We compared the localization accuracy using the radio-map generated by the proposed RGN and that measured by the walking survey. We used a radio-map constructed by randomly arranging APs to compare the localization accuracy according to the number of APs. The number of APs used was divided into 8, 12, 16, and 20 parts in consideration of the building environment.

**Figure 16:**
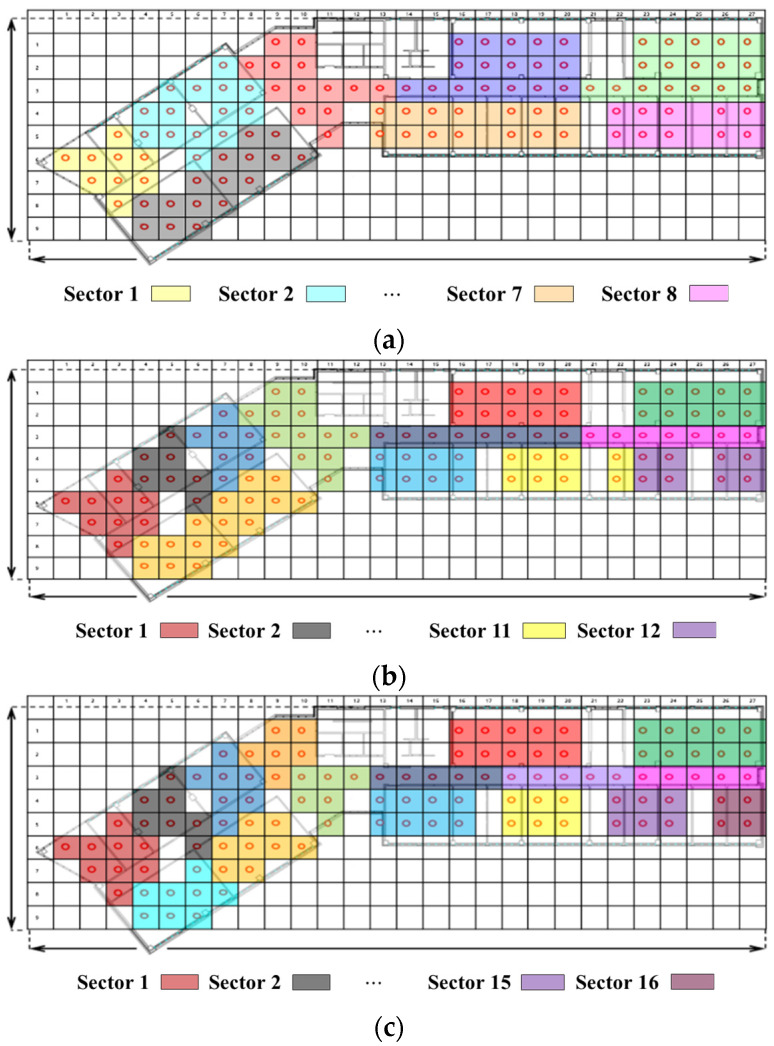

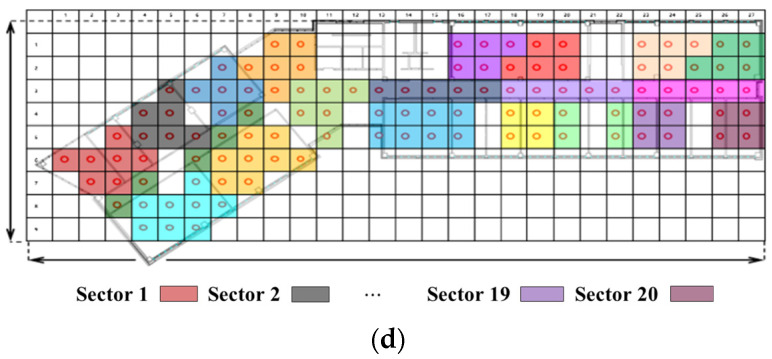
AP allocation partitions for each sector in the experimental environment divided by (**a**) 8, (**b**) 12, (**c**) 16, and (**d**) 20.

We divided the region map into sectors according to the number of APs to enable localization of all RPs. Figure 16 shows the sectors placed divided according to the number of APs in the region map of the localization experiment environment. We randomly selected one AP for each sector to compare and analyze localization performance. As for the experimental RSSI data, the RSSI data measured 200 times for each AP in RP were randomly sampled and converted to 250 for each RP, and the total number used in the experiment was 27,500.

**Figure 17:**
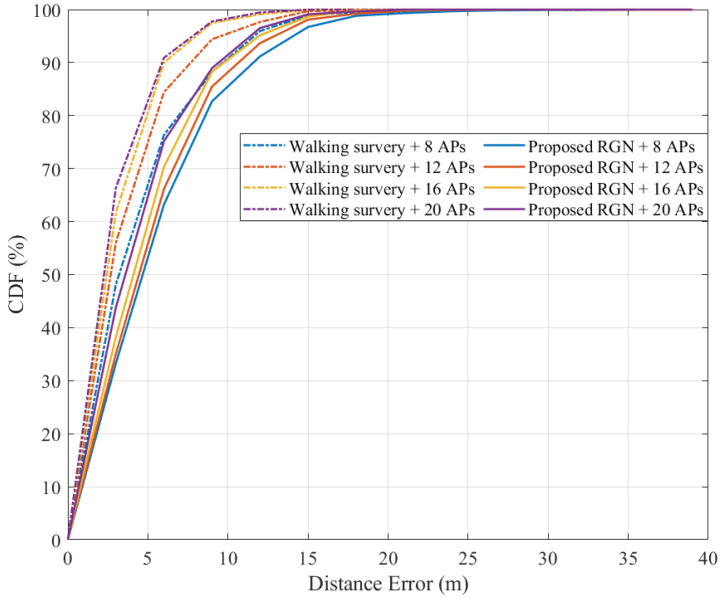
Comparison of CDF positioning errors between the proposed RGN and walking survey for varying numbers of installed AP.

Figure 17 shows the result of localization errors based on the radio-map constructed using the walking survey and the RGN in the cumulative distribution function (CDF) graph. There was a difference in performance according to the number of APs. As the number of installed APs increased from 8 to 20, the localization error decreased from 2.13 to 1.23 m in the walking survey and from 4.79 to 3.34 m in the proposed RGN. In the CDF graph, it can be seen that the walking survey using eight APs and the RGN performance using 20 APs were slightly different. 

**Figure 18:**
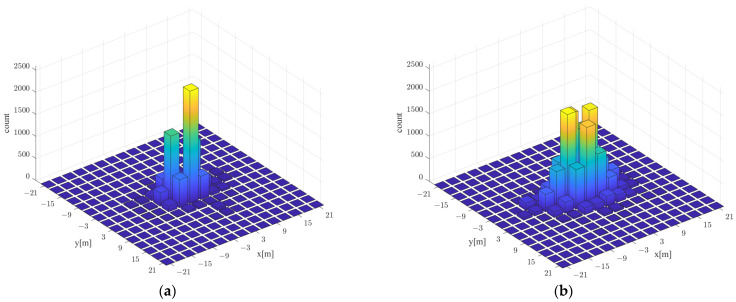
Distribution of localization error based on radio-map generated using (**a**) walking survey and (**b**) the proposed RGN.

Figure 18 shows the error distribution of real and predicted locations using walking survey and proposed RGN-based localization. The *x*-axis and *y*-axis represent the error location of each axis, and the z-axis represents the frequency according to the error of each predicted location. In this experiment, localization was performed 27,500 times using the experimental data randomly sampled 250 times among the RSSI data measured 200 times per AP at 110 locations in the environment shown in Figure 15d. The distribution of localization errors based on the walking survey shows that the maximum frequency was 2550 at the point where the *y*-axis was 3 m apart, which is biased compared with other points. The distribution of the proposed RGN-based localization errors was the maximum frequency of 1967, which showed a balanced form according to the direction. The two approaches had significant differences in localization performance, but the radio-map generated using the proposed RGN only required information on the floor plan, and the radio-map constructed using the walking survey required RSSI of each AP collected with 200 repetitions per RP. In other words, by implicitly inferring radio-map using the proposed RGN, there was an advantage of reducing collection times and workloads.

## 5. Conclusions

In this study, we proposed an APCW-based RGN to reduce the time and cost required to generate radio-maps and to enable the utilization of existing databases for fingerprinting. Conventional approaches required a person to collect RSSI or construct a radio-map using methods such as SLAM using a mobile robot. On the other hand, an APCW-based RGN can minimize labor and time because it can generate a radio-map without direct RSSI collection process through a human or mobile robot when there is data on the floor plan and radio-map of various structures. APCWs contained information needed for generating a radio-map, because they were created by converting obstacles present in the indoor space into data according to their materials and by partitioning them based on the AP location. The proposed RGN enabled significant reductions in the cost required to collect Wi-Fi signals of multiple buildings by generating radio-maps using an extant database. As a result of conducting radio-map generation experiments using an APCW-based RGN, an accuracy of 4.01 dBm was obtained when the size of the APCW was set to $39\times 39{\;m}^{2}$. This indicates that the proposed APCW-based RGN accurately generated a good radio-map, because the standard deviation of typical Wi-Fi signals fell within 5 dBm [45]. As a result of analyzing the weight vectors of the network trained using APCW, it was confirmed that the RGN successfully learned the propagation characteristics required for the accurate prediction of radio-maps. From these experiments, it was revealed that the RGN learned the configuration changes according to the indoor structural patterns and generated appropriate radio-maps. Since the experiment was performed by constructing a radio-map dataset in one building, the performance of the proposed APCW-based RGN when applied to other similar buildings is unknown. However, in the future, radio-maps are expected to be built on a floor plan composed of various structures because current technologies focus on research on how to collect RSSI data. In other words, by utilizing the sufficient training dataset of the previously constructed floor plan and radio-map, a radio-map can be generated without a direct RSSI collection process through a person or a mobile robot in a space constructed with a similar obstacle structure. Therefore, it is expected that the cost of generating radio-maps for fingerprint recognition technology in all buildings will be significantly reduced.

## Figures and Tables

**Table 1:** Variables used in the APCW-RGN.

Variables	Definitions
L	localization domain
S	Spatial information
$S_{\left(x,y\right)}$	Index values according to material types at x and y coordinates of L
$S_{x,y}^{i}$	One-hot vector according to index values at x and y coordinates of L
$L_{one-hot}$	localization domain encoded by one-hot vector
$APCW_{j}$	Access-point-centered window
d	Distance partitioned around the AP
W	Window size
${\bar{RSSI}}_{\left(x,\;y\right)}$	Normalization of $RSSI_{\left(x,y\right)}$
z	Latent variable
${ℒ}_{G}$	Loss function of generator in RGN
${ℒ}_{D}$	Loss function of discriminator in RGN

**Table 2:** Cell properties designed for obstacle Material.

Cell			
Index i	Object	Material	$\mathrm{One}-\mathrm{Hot}\;\mathrm{Vector}\;S^{i}$
0	Inaccessible Space	-	{0, 0, 0, 0, 0, 1}
1	Free Space	Vacuum	{0, 0, 0, 0, 1, 0}
2	Glass Wall	Glass	{0, 0, 0, 1, 0, 0}
3	Concrete Wall with Window	Concrete, Glass	{0, 0, 1, 0, 0, 0}
4	Concrete Wall	Concrete	{0, 1, 0, 0, 0, 0}
5	Concrete Wall with Iron Door	Concrete, Iron	{1, 0, 0, 0, 0, 0}

**Table 3:** Detail of Generator.

Network	Layer	Activation Function	Input Size	Output Size
Generator	FC	ReLU	$z+W^{2}\times i_{n}$	$2\times W^{2}$
	FC	ReLU	$2\times W^{2}$	$W^{2}÷3$
	FC	ReLU	$W^{2}÷3$	$W^{2}÷9$

**Table 4:** Detail of Discriminator.

Network	Layer	Activation Function	Input Size	Output Size
Discriminator	FC	ReLU	$W^{2}÷9+W^{2}\times i_{n}$	$W^{2}÷9$
	FC	ReLU	$W^{2}÷9$	$W^{2}÷27$
	FC	ReLU	$W^{2}÷27$	1

**Table 5:** Network Definition and Architecture.

Model	Description	Network	Layer	Input Size	Output Size
1	Floor plan without considering the material of the obstacle	Generator	FC(ReLU)	z+X×Y	X×Y÷9
		Discriminator	FC(ReLU)	X×Y÷9+X×Y	1
2	Floor plan considering the material of the obstacle	Generator	FC(ReLU)	$z+\left(X\times Y\right)\times i_{n}$	X×Y÷9
		Discriminator	FC(ReLU)	$X\times Y÷9+\left(X\times Y\right)\times i_{n}$	1
3	APCW without considering the material of the obstacle	Generator	FC(ReLU)	$z+W^{2}$	$W^{2}÷9$
		Discriminator	FC(ReLU)	$W^{2}+W^{2}÷9$	1
4	APCW considering the material of the obstacle	Generator	FC(ReLU)	$z+W^{2}\times i_{n}$	$W^{2}÷9$
		Discriminator	FC(ReLU)	$W^{2}÷9+W^{2}\times i_{n}$	1

**Table 6:** The Sizes of The RGN Models 1 to 4.

Model	Network	Layer	Size
1	Generator	FC(ReLU)	28,712,051
	Discriminator		79,138
2	Generator	FC(ReLU)	97,565,811
	Discriminator		79,138
3	Generator	FC(ReLU)	9,819,745
	Discriminator		38,307
4	Generator	FC(ReLU)	32,954,155
	Discriminator		38,307

**Table 7:** Accuracy of Radio-map Generation for Each Model.

Network	RMSE (dBm)		
	Average	Max	Min
Model 1	28.84	37.84	22.71
Model 2	5.48	7.06	4.1
Model 3	14.98	24.4	6.35
Model 4	4.01	6.43	2.26

## Data Availability

Not applicable.
